# On the Integration of Agents and Digital Twins in Healthcare

**DOI:** 10.1007/s10916-020-01623-5

**Published:** 2020-08-04

**Authors:** Angelo Croatti, Matteo Gabellini, Sara Montagna, Alessandro Ricci

**Affiliations:** Computer Science and Engineering Department (DISI), University of Bologna, via dell’Università, 50, Cesena, Italy

**Keywords:** Digital twin, Agents, MAS, Healthcare, Trauma management

## Abstract

A digital twin is a digital representation of a physical asset reproducing its data model, its behaviour and its communication with other physical assets. Digital twins act as a digital replica for the physical object or process they represent, providing nearly real-time monitoring and evaluation without being in close proximity. Although most of their concrete applications can be found mainly in the industrial context, healthcare represents another relevant area where digital twins can have a disruptive impact. The main research question tackled by this paper is about the integration of digital twins with agents and Multi-Agent Systems (MAS) technologies in healthcare. After providing an overview of the application of digital twins in healthcare, in this paper, we discuss our vision about agent-based digital twins, and we present a first case study, about the application of agent-based digital twins to the management of severe traumas.

## Introduction

The concept of *Digital Twin* (DT) originally appeared in manufacturing literature at the beginning of 2010s, referring to a digital representation of an asset (e.g., physical objects, processes, devices) containing the model of its data, its functionalities and communication interfaces [10]. A DT provides both the elements and the dynamics of how the asset operates throughout its life cycle, emphasising the connection between the physical model and the corresponding virtual counterpart [10].

In recent years, digital twins have become an important concept in Industry 4.0 [21], and in particular into the process of designing and developing industrial assets, involving different research fields—from the Internet of Things (IoT) to Simulation and Artificial Intelligence. A digital twin of an industrial asset collects in real-time data provided by the asset’s sensors to build a digital counterpart. Using this counterpart, it is possible carrying out simulations about the current and future state of the asset, reasoning about potential condition before they occur, or continuously collecting and analysing data on the ongoing state of the asset to prevent unwanted situations. This can be exploited for operation optimisation and for the maintenance of physical assets, systems and manufacturing processes.

Besides manufacturing and Industry 4.0, the digital twin paradigm is more and more permeating in other domains, and healthcare is a main one [4, 17, 24]. In healthcare, the DT paradigm is being explored for different purposes. An example is personalised medicine [1], exploring the use of digital twins as a dynamic digital replica of patients, created with historically available information. The digital twin, in this case, is meant to be useful for realising more effective care interventions, helping physicians and other intersecting care technologies in understanding the medical state of the patient. Another example is strategic planning: by creating a digital twin of a hospital, operational strategies or medical processes, it is possible to use the digital counterparts to determine what actions to take, eventually exploiting simulation facilities.

In this context, the main research question that we consider in this paper is about the fruitful integration of DT with agents and MAS in healthcare, in particular what sort of integration can be conceived and what are benefits that *agent-based DT* architectures can bring. Generally speaking, from the agent perspective, digital twins provide an effective blueprint for conceiving and designing digital environments mirroring the physical world, providing models to reason about them and support their decision making, and cooperation with human users as well. From the digital twin perspective, agents provide a blueprint for engineering intelligent systems – embedding AI and Distributed AI techniques, featuring some level of autonomy – on top of DTs, so that digital twin features could be exploited by e.g. personal assistant agents supporting medics in doing their work and cooperate.

In this paper, first we give a comprehensive background and survey on digital twins and their connection with the healthcare context (Section “Background”); then, we focus on the design of agent-based digital twins for healthcare (Section “Agent-based digital twins in healthcare”). As a concrete real-world case, we discuss the application of agent-based DTs to support the process of trauma management (Section “A case study: Trauma management”). We conclude the paper by sketching the challenges and the road ahead that we see about agent-based DTs (Section “The road ahead”).

## Background

**Fig. 1:**
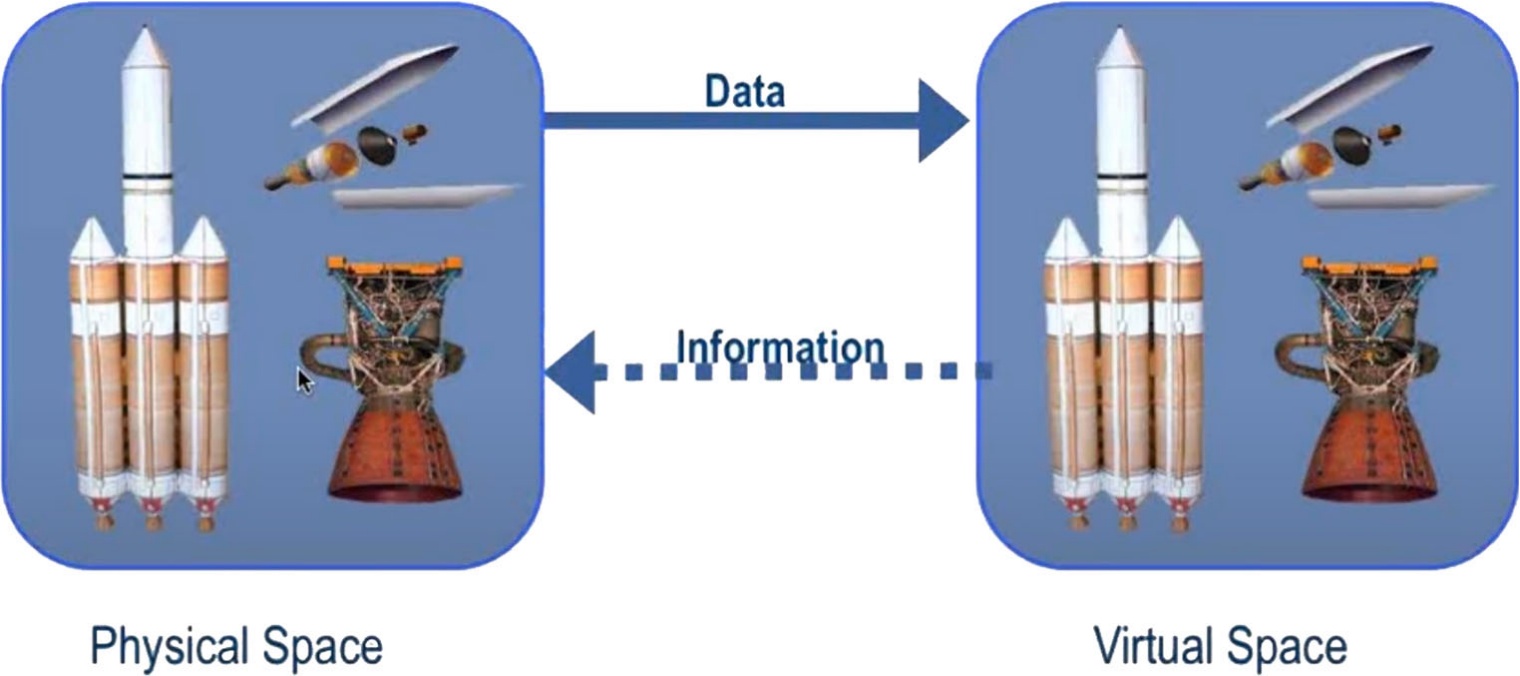
The Digital Twin model proposed in [9], based on three dimensions: physical, virtual, and connection

There is not a common agreement on the definition of DTs, especially because the concept has been evolved over the years, since its initial formulation in the aerospace field by the National Aeronautics and Space Administration (NASA) [8, 10]. In that original formulation, a DT is characterised by three dimensions: *physical*, *virtual*, and *connection*, as illustrated in Fig. 1, where the virtual space is mapped to the physical space through the connection part that exchanges information.

Our literature review brings to a set of characteristics that together define a DT. Everyone agrees that DT is a digital representation of the physical world that possibly includes models of the structure, functionalities and behaviour of the real counterpart [10, 16]. It can persist for the whole system life-cycle, and it is strongly linked with the physical entity: its state is continuously updated in near-real-time with data acquired on the physical system by different devices, mainly sensors or other sources such as existing IT systems (e.g. ERP, PLM), and transferred digitally [3]. The success of DTs and their wide application in Industry [22] is mainly due to the continuous interaction and connection between the real and digital worlds, that provide enormous benefits for the management and design of manufacturing industries: by adopting simulation or machine learning algorithms, they are used to make predictions on the evolutionary dynamic and future state of the real system, enabling to anticipate failures, to optimise the system, to design novel features, to ease and accelerate decision making, to improve productivity, just to cite some [23].

Besides industry, other domains that exploit Internet of Things technologies can benefit from the adoption of DTs. In this paper, we are particularly interested in the healthcare domain, where IoT has been already proved to impact healthcare services effectively [14, 15]. We envision that DTs can bring different advantages in this field, from supporting hospital organisation and management, for instance in medical pathway planning, in medical or asset resource allocation, to improving diagnostics, treatment and care, for instance predicting patient outcomes and disease progression or identifying personalised therapies.

The literature already refers to such applications which mainly exploit simulation, a tool already well known and adopted in the medical field. Figure 2 shows a representation of the DT concept applied to the healthcare context as it is currently envisioned in most of the literature and available industrial solutions. In particular, its introduction in healthcare is for modelling physical assets, i.e. a hospital, with the purpose to offer analytics and simulations capabilities. Liu et al. in [11] discuss the potential benefit of applying the concept of DT in healthcare and reports the case of CloudDTH, a framework based on DT healthcare. The case study presented aims at supporting self-management of elderly people, where CloudDTH provides real-time monitoring and enables personalised healthcare. In [12], the specific example of a Cardio-vascular DT is introduced. By replicating the physical system, it is used for the dual purpose of performing simulations that answer what-if questions, and of generating large scale synthetic data to be used to train machine learning algorithms. DTs of patients have also been introduced in [1], where they are adopted for identifying the best drug among the thousands possible to treat a certain disease. The vision is “Making mistakes on computer models instead of people”.^1^ There, a DT of a patient with symptoms of a specific disease is developed in unlimited copies, based on network models of all molecular, phenotypic, and environmental factors relevant to disease mechanisms. Simulations with different drugs are then performed to identify the treatment.

## Agent-based digital twins in healthcare

The idea of agent-based Digital Twins that we put forth in this paper has its root in the concept of *mirror worlds*, as originally introduced by David Gelernter [7] and more recently extended and developed in the context of Multi-Agent Systems [20]. A mirror world is a digital layer – operated by software agents – which is *bi-directionally coupled* with some physical environment, so that any relevant physical entities, including human users working in that context, have a digital counterpart in the mirror, that could be observed and acted upon by agents. This coupling between the digital and the physical layer can be exploited in order to design smart environments, where mirror worlds provide different kinds of *augmentations* for humans working/living there—namely cognitive, social, temporal augmentations [19].

**Fig. 3:**
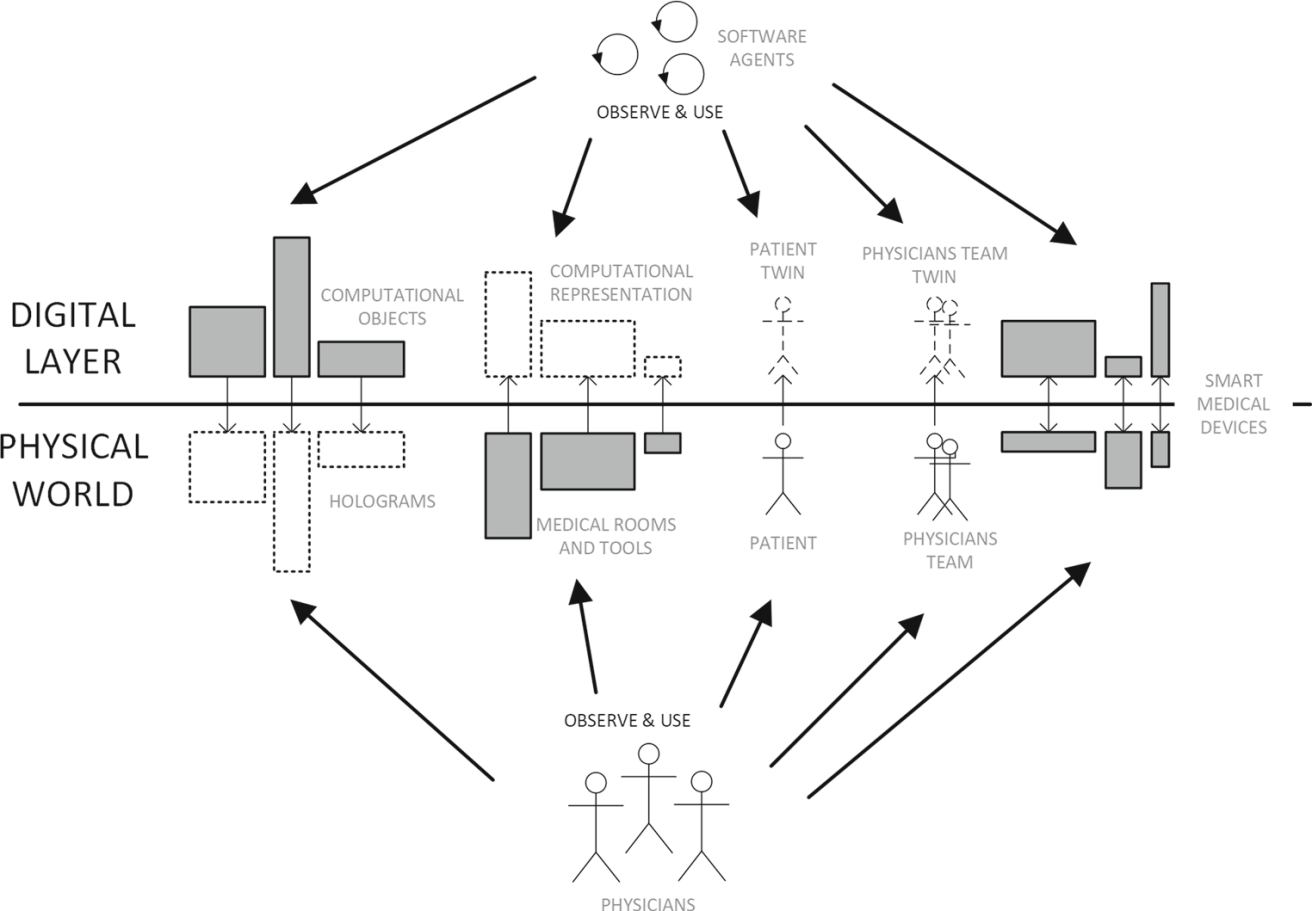
Agent-based digital twins as mirror worlds – a conceptual representation for the healthcare context

In that perspective, we see the DT as the enabling layer in healthcare for building agent-based smart environments shaped as mirror worlds (see Fig. 3). Any relevant assets of a healthcare context could have a digital counter-part – its twin – modelled as part of the environment that software agents can perceive and act upon. In so doing, the observable state of that digital twin perceived by the agents is coupled with the state of the physical twin, and the specific model adopted would depend on the desired level of abstraction. Physical assets modelled as part of the MAS environment could be either atomic, like e.g. a patient, a vital sign monitor device, or a vehicle, or composite, as a structure including the link to other, independent digital twins, such as a hospital including rooms, medics, patients, and so forth. Assets could refer not only to specific things but also *processes*, like the trauma management that will be discussed in Section “A case study: Trauma management”. This perspective makes it possible to explore the use of the simulation feature, which is brought by DT on the agent side, to support agent decision making. For example, the execution of simulations can be useful to agents for generating beliefs about states or events that could happen in the future, that may trigger or call for an agent’s action in the present. Or, as another example, an agent can check the effect that some planned action is going to have given the current state of the twin/asset, before committing to its execution.

**Fig. 4:**
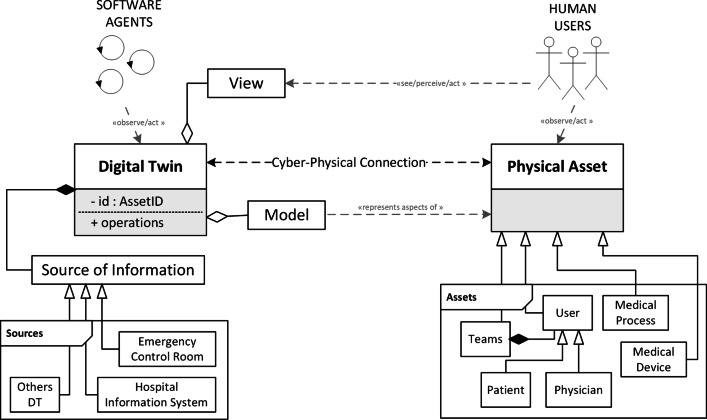
A meta-model for Agent-based Digital Twins in the healthcare context

Figure 4 shows the meta-model we designed to summarise main first-class concepts for agent-based digital twins in the healthcare context. In particular, the digital twin concept (identified with an id referring to the coupled physical asset) at this level of details exposes a *model* and a *view*. Viceversa, the (autonomous) control is encapsulated into the digital twin itself. On the one hand, the model represents for the DT all the features and aspects of the physical asset to which the DT is coupled through a cyber-physical connection, properly defined. On the other hand, the view allows humans to see and interact directly with the DT – e.g., through holograms or smart ICT systems. Agents, instead, can observe and interact with the digital twin exploiting digital twin’s operations – as well as humans can interact with the coupled physical asset. Finally, the digital twin could also communicate directly with external sources of information, i.e. other software systems, sensors, and so on. Other digital twins could act as a source of information as well for a particular DT. Considering the particular context of application, in the figure, we also reported possible examples of the source of information and even examples of potential involved physical assets that could benefit from a digital twin oriented scenario. In particular, among them, it is worth clarifying that not only real things – such as medical devices – can represent a physical asset. Also, users (including patients and physicians) and medical processes can be considered physical assets for a digital twin representation.

## A case study: Trauma management

In this section, we describe a first case study^2^ where an agent-based digital twin is being designed to digitalise and support the process of severe traumas management.

### The trauma context

Among the time-dependent pathologies, trauma management is the most critical one. When trauma occurs, we can observe two process’ main phases: the *pre-hospital phase* – when the patient is reached by a physician on the accident place to provide to him/her first aid and transferred to the hospital emergency department – and the consequent *operative phase* – when the patient is assisted by the trauma team within the hospital emergency department. In the following, we will refer to these two phases as *PreH* and *Trauma*, respectively.

A main important action in trauma management is the collection of the trauma documentation: a document reporting everything occurred to the patient during the trauma management (procedures, administered drugs, diagnostics reports, vital signs trace, and so on) must be produced. Trauma documentation is essential for a posteriori analysis but also when trauma management is ongoing. In this case, the trauma leader – the physician who leads the trauma team – can always have a comprehensive general look on the ongoing trauma for a better decision on how to proceed in order to save patient’s life. Many other details about the application of smart technologies to assist the trauma team in producing trauma documentation can be found in our previous works [5, 6, 13] where we describe the TraumaTracker project, a PMDA (Personal Medical Digital Assistant) designed and developed for this specific purpose.

After two years experimenting the system (in which about 800 trauma reports have been collected), a further important desiderata emerged, that is: the need of a continuous monitoring of the complete state of the trauma, of the involved patient and care team even in the pre-hospital phase. This lead to the goal of re-engineering the TraumaTracker system so as to adopt an agent-based digital twin architecture across the two main phases.

### An agent-based digital twin for the trauma management process

**Fig. 5:**
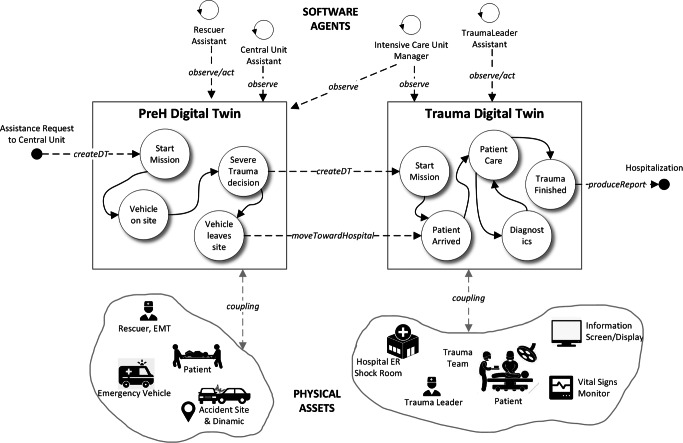
A conceptual representation of the involved digital twins for the trauma management process

The basic idea is to consider the trauma management process as a physical asset which is suitably mirrored by two digital twins, digitalising the process related to the PreH phase (PreH digital twin) and the process related to the management of the trauma inside the hospital (Trauma digital twin). Figure 5 shows a conceptual representation of this design. This architectural choice follows the real evolution of trauma management. It is in the PreH phase that rescuers take in charge the case of the patient and decide if either the current situation is a severe trauma or not, and only in the former case the trauma team (and the second phase) is triggered. The physical assets and software agents in the two cases are different (see Fig. 5).

#### The PreH digital twin

This digital twin represents the digital counterpart of the pre-hospital care process. Abstracting from details, the digital twin instance starts when the rescue central unit receives the call for assistance. The model of this digital twin involves:

- the *vehicle*, sent to the accident site with the rescuers;
- the EMT (emergency medical technician) and the *rescuer*;
- the *accident place* and, in particular, the *accident dynamic*;
- the *patient*.

This digital twin collects real-time information considering the information given by the central unit, the GPS System of the vehicle, and the smart devices held by rescuers to compile emergency forms. During its life-cycle, the most relevant moment is related to the transition to the state where rescuers decide the degree of severity of the ongoing trauma (considering patient’s health state and its GCS—Glasgow Coma Scale value).

#### The trauma digital twin

This digital twin represents the operative phase of trauma management and starts when, in the previous phase, the trauma is marked as severe. The fact that this digital twin starts before the arrival of the patient to the hospital is very important for this case study. In this way, the trauma team is pre-alerted about the incoming patient and start to collect and receive information directly from the accident site. Its internal state changes when the patient is delivered to the emergency department, that is, when the trauma team starts in taking care of the patient. The model of this digital twin involves the following assets:

- the *patient* and all information flow coming from *connected devices* (i.e., the vital signs monitor);
- the *trauma leader*, and the trauma team members;
- the *shock-room*, the room of the emergency department where generally each trauma is managed;
- other tools and equipment (e.g., rapid diagnostics machineries, displays to show real-time information of the ongoing trauma, and so on).

Some data composing the internal state of this digital twin come from the previous digital twin, others are collected by the coupled assets. Its life-cycle contemplates the macro-steps related to trauma management, terminating when the patient is ready for the hospitalisation.

#### Software agents

From the agent perspective, the full digital twin system is observed and accessed by several agents acting as personal assistants of involved professional figures or as managers of involved rooms and places. For instance, the agent acting as a personal assistant of the trauma leader, assists this physician for the trauma documentation, updating the digital twin state considering the ongoing performed procedure and administered drugs. Agents behave according to the digital twin state, updating their belief upon it.

### Current implementation

A very preliminary version of a system prototype has been developed according to the designed conceptual model. It’s architecture is service-oriented (SOA):^3^ each digital twin is developed as a micro-service exposing an ad-hoc RESTful API to access to data and information of the digital twin. Each micro-service has been developed using the Java programming language and the Vert.x library.^4^ Security aspects and access control are mediated by the hospital local area network where the whole system is deployed. The digital twin microservices are in execution on the hospital private cloud infrastructure and can be accessed only from applications running within such context. Software agents have been designed according to the A&A meta-model [18] exploiting the JaCaMo framework [2] for developing Multi-Agent Systems (MAS). Future exploration will consider using JaCaMo also to develop (part of) the micro-services composing the digital twins.

## The road ahead

Healthcare represents an application domain where the introduction of digital twins could have a disruptive impact. In this area, digital twins could be the digital counterpart not only for physical computational assets (e.g., vital signs monitors, diagnostic machinery, surgery rooms, etc.) but also for care processes—from the simplest to the most critical ones, such as the trauma management used as a case study in this paper.

In this paper, we put forth and discuss the idea of agent-based digital twins, integrating the digital twin paradigm with agents in a modelling and design framework based on mirror worlds. The main items of the research roadmap that we see for further developing this vision include: the investigation of DT semantic models effective in supporting agent reasoning about DT—eventually based on existing standards and efforts such as semantic web; understanding how agent-based digital twins could be used to devise the architecture of future healthcare information systems, beyond current standards such as the ISO 12967;^5^ and the investigation of simulation as brought by DT to support agent decision making.

Finally, the introduction of digital twin paradigm also involves a discussion on related ethical aspects, especially when its application context is the healthcare one. Such a discussion is out of the scope of this paper, but future work will be devoted to taking into the account also this issue. It is clear that a digital twin perspective could influence people work and decisions, in particular in such contexts where decisions must be rapidly taken, such as time-dependent healthcare procedures.

## Footnotes

1 https://www.digitwins.org/

2 This case study is currently under development in collaboration with the Trauma Center of the “M. Bufalini” Hospital in Cesena (Italy), the hub for traumas occurring on the Romagna area of Italy.

3 http://www.soa-manifesto.org

4 https://vertx.io/

5 https://www.iso.org/standard/50500.html
